# Loratidine is associated with improved prognosis and exerts antineoplastic effects via apoptotic and pyroptotic crosstalk in lung cancer

**DOI:** 10.1186/s13046-023-02914-8

**Published:** 2024-01-02

**Authors:** Xiwen Liu, Ran Zhong, Jiaxing Huang, Zisheng Chen, Haoxiang Xu, Lixuan Lin, Qi Cai, Miao He, Shen Lao, Hongsheng Deng, Caichen Li, Jianfu Li, Yongmei Zheng, Xiaoyan Liu, Riqi Zeng, Jianxing He, Wenhua Liang

**Affiliations:** 1 Department of Thoracic Surgery and Oncology, the First Affiliated Hospital of Guangzhou Medical University, Guangzhou, China; 2 China State Key Laboratory of Respiratory Disease & National Clinical Research Center for Respiratory Disease, Guangzhou, 510120 China; 3 Department of Respiratory and Critical Care Medicine, The Sixth Affiliated Hospital of Guangzhou Medical University, Qingyuan People’s Hospital, Qingyuan, 511500 China; 4 The Second Affiliated Hospital (Guangdong Provincial Hospital of Chinese Medicine), Guangzhou University of Chinese Medicine, Guangzhou, 510120 China; 5 School of Clinical Medicine, Henan University, Kaifeng, 475000 China; 6 Nanshan School, Guangzhou Medical University, Jingxiu Road, Panyu District, Guangzhou, 511436 China; 7 Southern Medical University, Guangzhou, 510120 China; 8 The First People’s Hospital of Zhaoqing, Zhaoqing, 526000 China

**Keywords:** Lung cancer, Loratadine, Apoptosis, Pyroptosis, Caspase8

## Abstract

**Background:**

Tumor-associated inflammation suggests that anti-inflammatory medication could be beneficial in cancer therapy. Loratadine, an antihistamine, has demonstrated improved survival in certain cancers. However, the anticancer mechanisms of loratadine in lung cancer remain unclear.

**Objective:**

This study investigates the anticancer mechanisms of loratadine in lung cancer.

**Methods:**

A retrospective cohort of 4,522 lung cancer patients from 2006 to 2018 was analyzed to identify noncancer drug exposures associated with prognosis. Cellular experiments, animal models, and RNA-seq data analysis were employed to validate the findings and explore the antitumor effects of loratadine.

**Results:**

This retrospective study revealed a positive association between loratadine administration and ameliorated survival outcomes in lung cancer patients, exhibiting dose dependency. Rigorous in vitro and in vivo assays demonstrated that apoptosis induction and epithelial-mesenchymal transition (EMT) reduction were stimulated by moderate loratadine concentrations, whereas pyroptosis was triggered by elevated dosages. Intriguingly, loratadine was found to augment PPARγ levels, which acted as a gasdermin D transcription promoter and caspase-8 activation enhancer. Consequently, loratadine might incite a sophisticated interplay between apoptosis and pyroptosis, facilitated by the pivotal role of caspase-8.

**Conclusion:**

Loratadine use is linked to enhanced survival in lung cancer patients, potentially due to its role in modulating the interplay between apoptosis and pyroptosis via caspase-8.

**Supplementary Information:**

The online version contains supplementary material available at 10.1186/s13046-023-02914-8.

## Introduction

Lung cancer, with an incidence rate of 11.4% and a mortality rate of 18%, significantly threatens human survival and social development [1]. The complex relationship between inflammation and tumor progression suggests that inflammatory microenvironments could either prevent or promote tumor formation. On the one hand, chronic inflammation shares similarities with cancer, such as increased angiogenesis and apoptosis inhibition [2]. On the other hand, it may prevent tumors by enhancing immune surveillance resulting from an immune response [3, 4]. Studies have shown that anti-inflammatory and antioxidant treatments may prevent or delay cancer onset and development. Nonsteroidal anti-inflammatory drugs, such as aspirin, metformin, and statins, have anticancer effects, along with other cell and inflammatory factor inhibitors [5, 6]. Recent research has begun to explore the potential of antihistamines in cancer treatment.

Antihistamines, functioning as antagonists or inverse agonists of histamine receptors, are employed in the management of conditions such as allergic rhinitis, allergic conjunctivitis, and urticaria [7]. Recent investigations have posited a potential application of antihistamines such as cimetidine, terfenadine and astemizole in the realm of cancer therapy [8–11]. For instance, some researchers observed that histamine can prevent the proliferation of a diverse array of cancerous cells, suggesting that antihistamines may serve as viable inhibitors of tumor expansion [12]. Shah and Stonier proposed that the influence of these drugs on cellular growth, apoptosis, and angiogenesis may confer anticancer properties [13]. Additional researchers have documented associations between antihistamine utilization and decreased risk of colorectal, breast, and lung cancers, underscoring the need for further exploration of the potential advantages of antihistamines in cancer prevention and management [14, 15].

The aforementioned studies offer an expanded perspective on the possible implications of antihistamines in cancer therapy. However, their exact therapeutic potential in Asian people and the most effective approaches for incorporating them into cancer treatment regimens remain to be clarified. As a result, our study aims to examine the anticancer mechanisms of loratadine, a commonly employed antihistamine, by employing Cox regression models to identify potential associations between noncancer drug exposure and patient prognosis. To corroborate our findings from the retrospective analysis and investigate the antitumor efficacy of the candidate agent, we conducted observations utilizing cultured cells, various animal models, and computational assessments of RNA-seq data.

## Results

### Patients receiving loratadine have better survival

Our study included 4522 patients, with 529 deaths (9.7%) during follow-up. Adenocarcinoma was the most common type (78.1%). Loratadine was used by 1299 patients (28.7%), with higher usage in the living group. Of the entire cohort, 41.1% were prescribed ipratropium bromide, 9.6% fluconazole, 6.9% loratadine, 11.3% ranitidine, 9.5% vitamin B6, 14.2% riboflavin sodium phosphate, 14.2% myrtle oil enteric-coated capsules, 1.3% felodipine, 4.0% cefetamet ivoxil, and 11.3% vitamin B6. Significant differences in medication usage, except for felodipine, were observed between the living and deceased groups (Table 1).

**Table 1:** Clinicopathological characteristics of patients

Variables	level	Overall	Alive	Death	*P* value
N		4522	3993	529	
Age (mean (SD))		58.77 (10.65)	58.42 (10.73)	61.39 (9.70)	< 0.001
Sex (%)	Male	2540 (56.2)	2148 (53.8)	392 (74.1)	< 0.001
	Female	1982 (43.8)	1845 (46.2)	137 (25.9)	
Smoke (%)	No	2393 (52.9)	2205 (55.2)	188 (35.5)	< 0.001
	Yes	1401 (31.0)	1165 (29.2)	236 (44.6)	
	Unknown	728 (16.1)	623 (15.6)	105 (19.8)	
Marital status(%)	Married (including common law)	4355 (96.3)	3843 (96.2)	512 (96.8)	0.596
	Divorced	4 (0.1)	4 (0.1)	0 (0.0)	
	Single (never married)	58 (1.3)	54 (1.4)	4 (0.8)	
	Unknown	105 (2.3)	92 (2.3)	13 (2.5)	
Histologic_Type_ICD_O_3 (%)	Adenocarcinoma	3530 (78.1)	3188 (79.8)	342 (64.7)	< 0.001
	Adenosquamous	63 (1.4)	44 (1.1)	19 (3.6)	
	Large cell carcinoma,	25 (0.6)	18 (0.5)	7 (1.3)	
	Neuroendocrine cancer	54 (1.2)	44 (1.1)	10 (1.9)	
	Non-small cell lung cancer	10 (0.2)	9 (0.2)	1 (0.2)	
	Sarcomatoid carcinoma	47 (1.0)	35 (0.9)	12 (2.3)	
	Signet ring cell carcinoma	64 (1.4)	45 (1.1)	19 (3.6)	
	Small cell carcinoma	621 (13.7)	510 (12.8)	111 (21.0)	
	Squamous cell carcinoma	1 (0.0)	1 (0.0)	0 (0.0)	
	Undifferentiated carcinoma	107 (2.4)	99 (2.5)	8 (1.5)	
Grade (%)	other	503 (11.1)	496 (12.4)	7 (1.3)	< 0.001
	Grade IModerately differentiated; Grade II	1538 (34.0)	1362 (34.1)	176 (33.3)	
	Poorly differentiated; Grade III	1103 (24.4)	906 (22.7)	197 (37.2)	
	Undifferentiated; anaplastic; Grade IV,	7 (0.2)	5 (0.1)	2 (0.4)	
	Unknown	1371 (30.3)	1224 (30.7)	147 (27.8)	
Loratadine use	No	3223 (71.3)	2812 (70.4)	411 (77.7)	
	Use	1299 (28.7)	1181 (29.6)	118 (22.3)	0.001

**Fig. 1:**
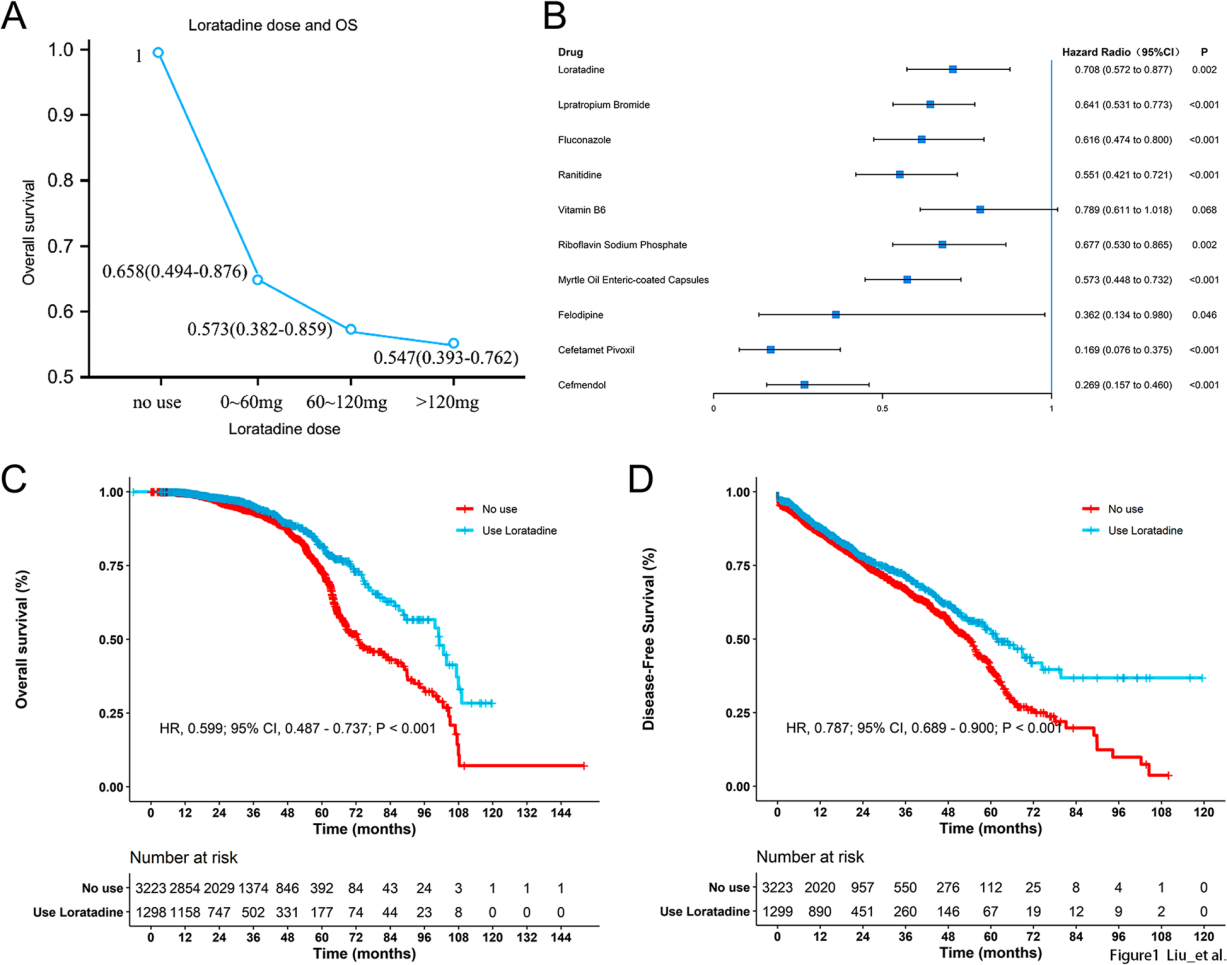
Uptake of loratadine is correlated with better survival in patients diagnosed with lung cancer. **A** Lung cancer mortality decreased in a dose-dependent manner with the increasing cumulative use of loratadine. **B** Hazard ratio for different drugs in the univariate analysis of OS. **C**-**D** The overall survival curve and disease-free survival curve with and without loratadine use

Following both univariate and multivariate analyses, significant associations with lung cancer overall survival (OS) were observed for loratadine (aHR 0.708, 95% CI, 0.572–0.877), ipratropium bromide (aHR 0.641, 95% CI, 0.531–0.773), fluconazole (aHR 0.616, 95% CI, 0.474–0.8), ranitidine (aHR 0.551, 95% CI, 0.421–0.721), Vitamin B6 (aHR 0.789, 95% CI, 0.611–1.018), Sodium Riboflavin Phosphate (aHR 0.677, 95% CI, 0.53–0.865), Standard Myrtle Oil, Dissolved Capsules (adult + size) (aHR 0.573, 95% CI, 0.448–0.732), the use of felodipine (aHR 0.362, 95% CI, 0.134–0.98), cefetamate (aHR 0.169, 95% CI, 0.76–0.375), and cefamandole (aHR 0.269, 95% CI, 0.157–0.46) were all significantly associated with lung cancer OS (Fig. 1B). However, only loratadine (aHR 0.859, 95% CI, 0.748–0.987), felodipine (aHR 0.706, 95% CI, 0.426–1.17), cefetamet (aHR 0.594, 95% CI, 0.421–0.838), and cefamandole (aHR 0.453, 95% CI, 0.302–0.679) maintained significant associations with lung cancer DFS (Fig. 1B and Table 2).

**Table 2:** Hazard ratio for univariate analyses of DFS in different drugs

Drug use	aHR (95% CI)
Loratadine	0.859(0.748–0.987)
Lpratropium romide	1.021(0.897–1.162)
Fluconazole	1.153(0.947–1.403)
Ranitidine	1.314(1.099–1.572)
Vitamin B6	1.256(1.064–1.483)
Riboflavin Sodium Phosphate	1.017(0.853–1.213)
Myrtle Oil Enteric-coated Capsules	0.894(0.748–1.069)
Felodipine	0.706(0.426–1.17)
Cefetamet Pivoxil	0.594(0.421–0.838)
Cefmendol	0.453(0.302–0.679)

The concomitant use of loratadine and other medications exhibited a reduced mortality risk compared to the administration of these drugs individually (Fig. E1). A dose-dependent relationship was observed between cumulative loratadine consumption and a decline in lung cancer mortality (*p* = 0.015 for trend test, Fig. 1A). Additionally, significant disparities were detected in the OS and PFS curves when comparing patients with and without loratadine treatment (Fig. 1C-D).

Taking into account the impact of chemotherapy on drug utilization, lung cancer risk, and mortality, the magnitude of the association between drug use and OS or DFS exhibited minor variations in the stratified analysis based on chemotherapy. Nevertheless, the direction of the association remained consistent with the overarching findings. In light of potential toxic side effects, loratadine was chosen as the drug of focus for this investigation.

### Moderate doses of loratadine may induce cell senescence and apoptosis while inhibiting epithelial-mesenchymal transition (EMT)

Loratadine was tested to discover the potential therapeutic mechanism in vitro and in vivo. First, the IC50 values of lung cell lines were calculated according to the results of CCK8 assays (Fig. E2).

**Fig. 2:**
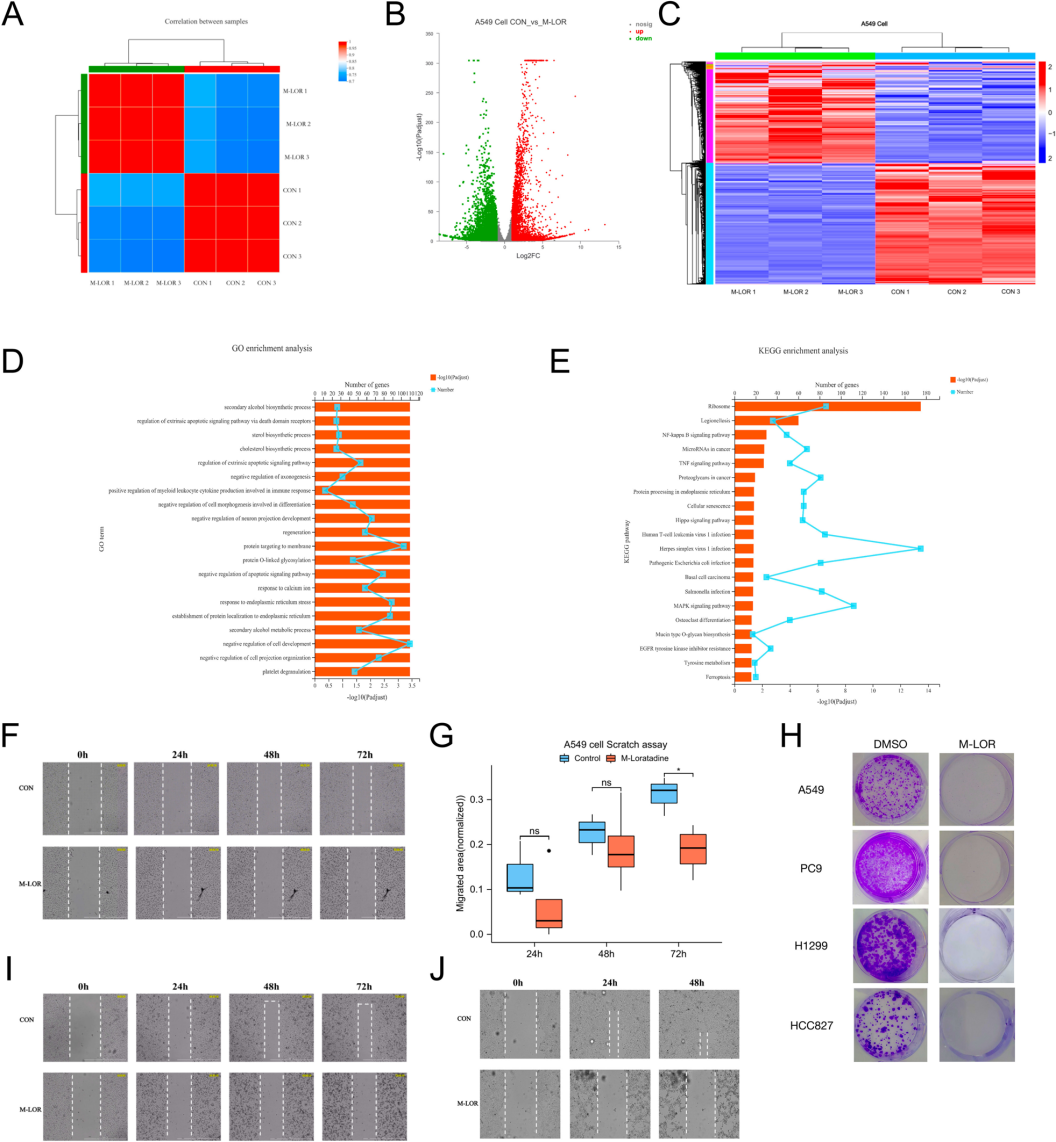
RNA sequencing of loratadine treatment in vitro. **A** PCA of RNA sequencing samples. **B** A549 cells were activated with loratadine (IC50) for 2 days (*n* = 3 biologically independent samples per group). RNA sequencing was performed. Differential gene expression is shown in a volcano plot. **C** A549 cells were activated with loratadine (IC50) for 2 days (*n* = 3 biologically independent samples per group). RNA sequencing was performed. Differential gene expression is shown in the heatmap. **D** GO enrichment categories of DEGs. **E** KEGG enrichment categories of DEGs. **F** Wound healing assay and live cell imaging of A549 cells. **G** Each column represents the mean value of the migrated area of the two different groups in A549 cells, and error bars indicate SD. *, *p* < 0.05. **H** Cell growth was examined by colony formation assays in various lung cancer cell lines. **I** Wound healing assay and live cell imaging of PC9 cells. **J** Wound healing assay and live cell imaging of HCC827 cells

For in-depth analysis, we treated A549 cells with moderate loratadine concentrations (IC50) or DMSO (as a control to exclude effects of the solvent) for 48 h and then performed RNA sequencing (RNAseq) analysis. Principal component analysis (PCA), volcano plot analysis and heatmap of RNA-sequencing data separated loratadine-treated from control groups (Fig. 2A-C). Critically, enrichment analysis performed on the differentially expressed genes revealed many overlapping processes, particularly those related to cell senescence and negative regulation of the vascular endothelial cell proliferation signaling pathway (Fig. 2D and E, Figs. E3 and E4).

**Fig. 3:**
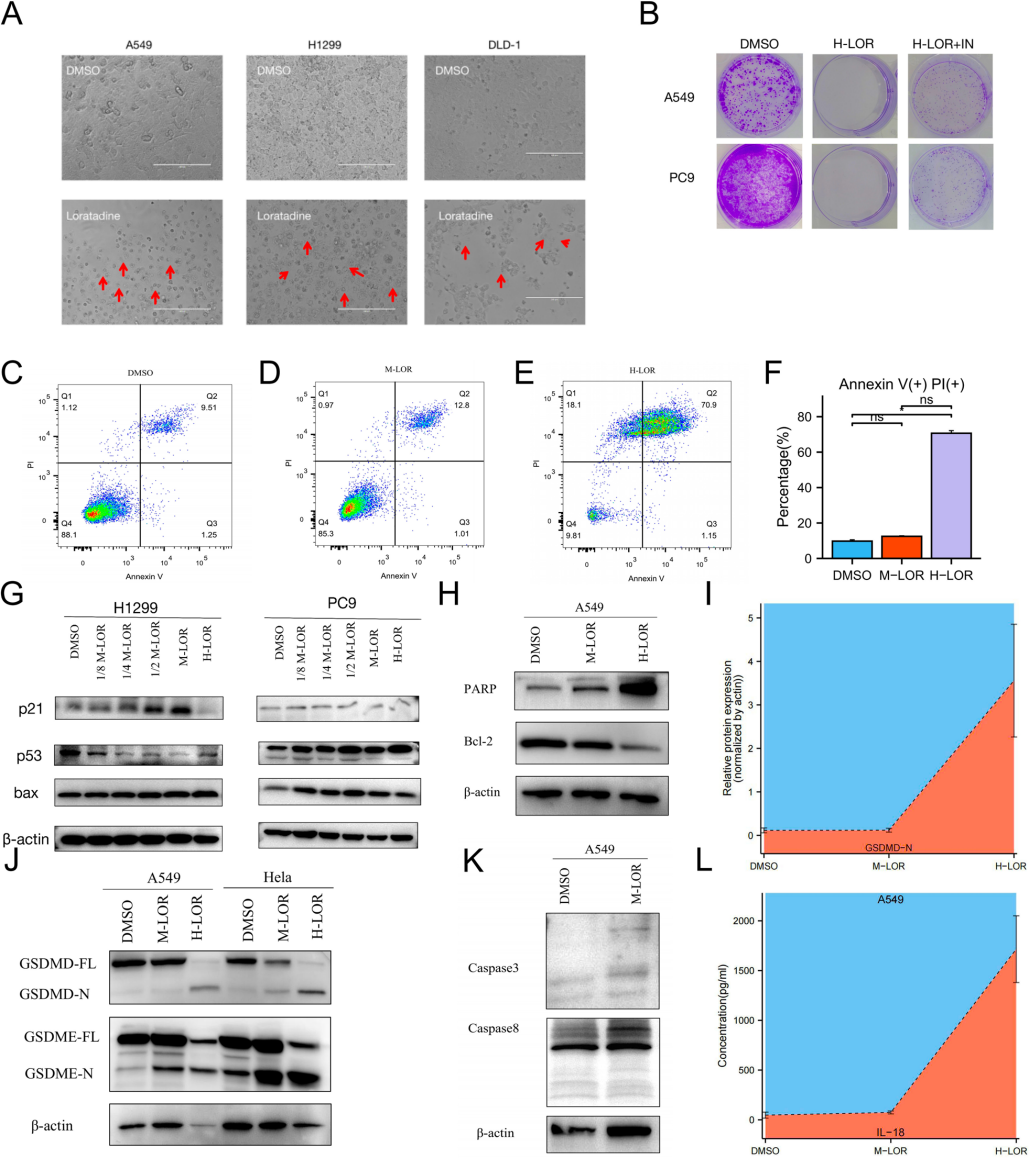
High-dose loratadine promoted the crosstalk between apoptosis and pyroptosis. **A** Evident balloon-like morphological changes were noted with red arrows in different cell lines treated with H-loratadine. **B** Cell growth was examined by colony formation in lung cancer cell lines treated with H-loratadine and combined with a GSDMD inhibitor. **C** to **F** Flow cytometry analysis of inhibitor-treated A549 cells stained with Annexin V-FITC and PI. The percentage of double-positive cells was presumably pyroptotic cells. **G**-**K** A549, PC9, and NCI-H1299 cells were treated with different doses of loratadine. The indicated proteins were analyzed by Western blotting. Dose-dependent cleavage of caspases, p53, p21, bax, PARP, GSDMD and GSDME was demonstrated. GSDMD-FL, full-length GSDMD; GSDMD-N, GSDMD N-terminal domain. GSDME-FL, full-length GSDME; GSDME-N, GSDME N-terminal domain. **L** Release from A549 cells treated with H-loratadine. Each column represents the mean value of three biological replicates, and error bars indicate SD. ***p < 0.001

**Fig. 4:**
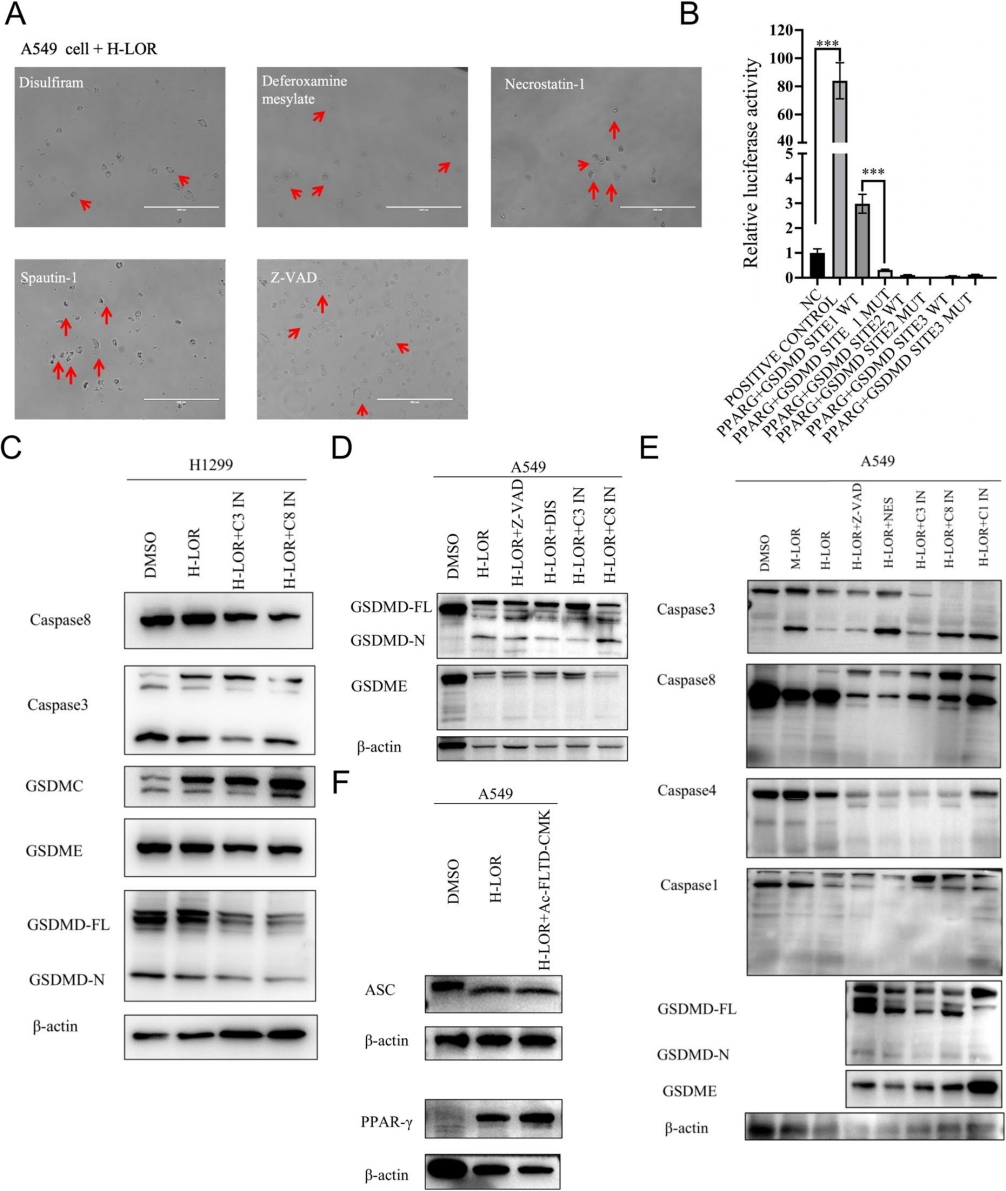
Pharmacological agents and caspase inhibitors partially reversed loratadine-induced cell death. A Pyroptotic cell changes, such as cell swelling and membrane blebbing, were noted with red arrows in A549 cells treated with H-loratadine as well as different cell death inhibitors. **B** Validation of PPARG as a promoter of GSDMD expression using a luciferase reporter assay. The promoter region of GSDMD was cloned upstream of the luciferase gene in the pGL3-Basic vector. The PPARG expression vector (vec PPARγ) was used to upregulate PPARγ expression. Bar graph representing relative luciferase activity in cells. Luciferase activity was normalized to Renilla luciferase activity for each sample. Data are presented as the mean ± SD of three independent experiments. ***P < 0.001 compared to control. **C**-**D** A549 and NCI-H1299 cells were treated with different doses of loratadine combined with cell death inhibitors or caspase inhibitors. The indicated proteins were analyzed by Western blotting. GSDME-FL, full-length GSDME; GSDME-N, GSDME N-terminal domain; C1 IN, caspase 1 inhibitor (ac-YVAD-cmk); C3 IN, caspase 3 inhibitor (z-DEVD-fmk); C8 IN, caspase 8 inhibitor (z-IETD-fmk); Z-VAD pan caspase inhibitor (z-VAD-fmk); M-LOR, moderate dose of loratadine; H-LOR, high dose of loratadine

To gain insight into the mechanism, we built a loratadine-targeted gene set based on the CTD mentioned above, of which genes were differentially expressed in the two groups (Fig. E5A). Subsequently, GO and KEGG enrichment analyses based on loratadine-targeted gene expression were further explored (Fig. E5B-G). Interestingly, the cell senescence signaling pathway was also enriched. In addition, pathways including the cell cycle, P53 signaling pathway and apoptosis were identified. Among them, cell senescence and apoptosis are frequently prevented by epithelial-mesenchymal transition (EMT) [16], which is known to broadly regulate cancer invasion and metastasis. As active caspase‐3 and caspase‐8 have been reported to enhance apoptosis, we therefore assessed whether loratadine induced apoptosis by evaluating caspase‐3/8 activity and BLC-2 reduction. Western blot analysis revealed increased cleavage of caspase‐3, caspase‐8, Bax, and PARP, while Bcl-2 levels decreased in loratadine-treated cells compared to the control group (Fig. 3G and H). Moreover, we carried out a scratch wound assay and live cell imaging of A549 (Fig. 2F, movies 1 and 2), PC9 (Fig. 2I, movies 3 and 4), and HCC827 cells (Fig. 2J, movies 5 and 6) as a functional test for cell migration and cell status in vitro. As indicated in Fig. 2G, drug treatment notably reduced the migratory ability of A549 cells in the 72-h group. The average level in the loratadine group was lower than that in the control group, with a difference of -0.124 (-0.221—-0.026), and the difference was statistically significant (t = -3.247, *P* = 0.023). Loratadine treatment also impacted colony formation, resulting in an 80–90% reduction compared to the control group (Fig. 2H).

**Fig. 5:**
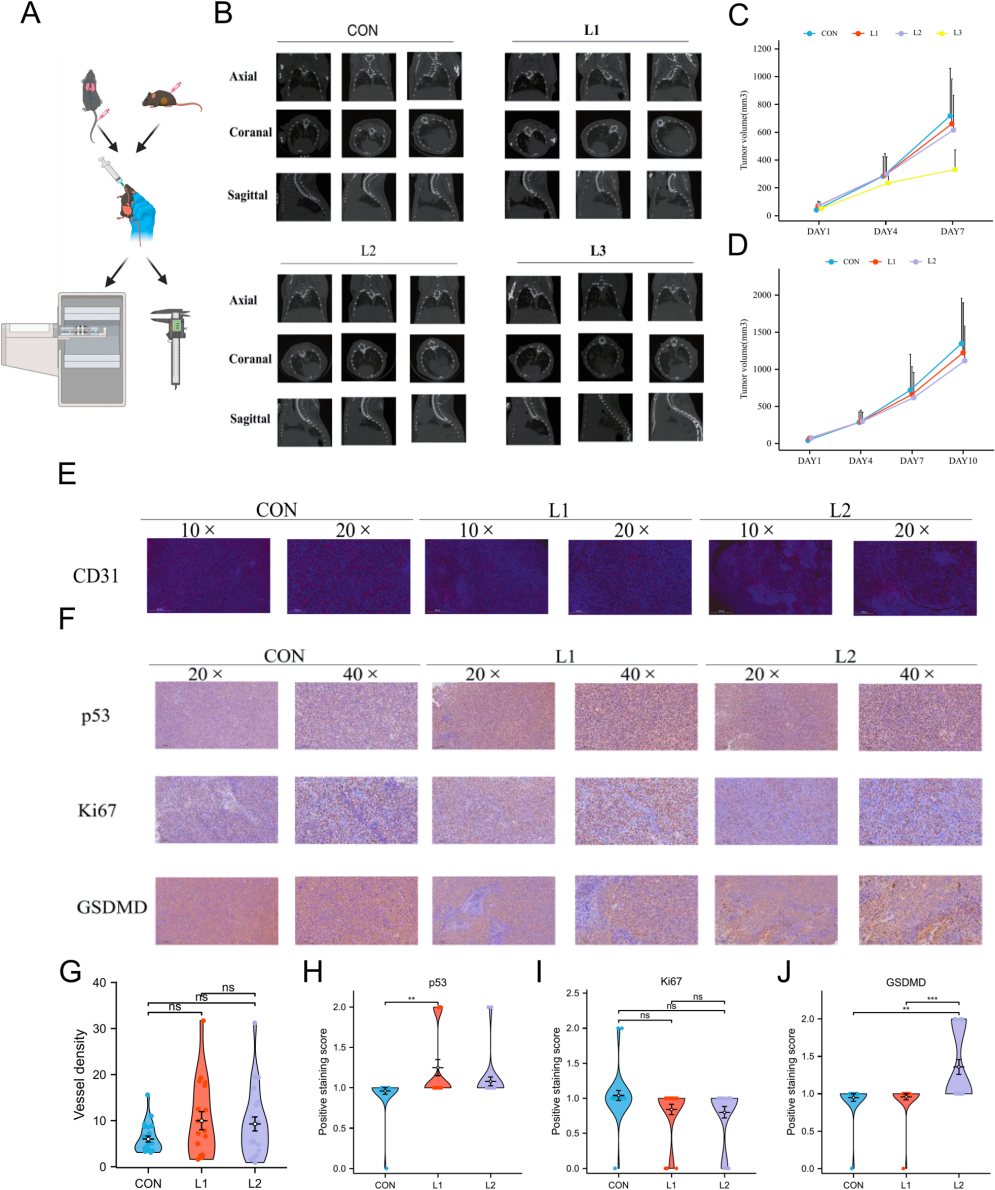
Loratadine treatment suppresses tumorigenesis in vivo. **A** Schematic overview of the mouse experimental model protocol. **B** Micro-CT images of lungs from mice following LLC tail vain injection with or without loratadine treatment. Three-dimensional rendering of micro-CT data with lungs in gray; the lost part represents the tumor. **C**-**D** The indicated cells were subcutaneously injected (0.5–1 × 10.^6^ cells per mouse) into C57BL/6 mice (*n* = 5). Tumor formation was analyzed. CON, vehicle group; L1, 5 mg/kg/d resveratrol group; L2, 35 mg/kg/d resveratrol group; and L3, 175 mg/kg/d resveratrol group. After 7 days of treatment, 4 mice died in the L3 group. **D** Vascular morphology of mouse subcutaneous tumor sections stained with DAPI (blue) and CD31 (red). Magnification: IHC 10 × and 20 × , Scale bar: 500 µm and 200 µm. **E** Tumor sections stained with p53, Ki67 and GSDMD. Magnification: IHC 20 × and 40 × , Scale bar: 200 µm and 100 µm. **F** Each violin plot represents the quantification of vascular density. ns, no significant difference. **G**-**I** Each violin plot represents the relative expression levels of each tumor section protein. Data are presented as the means ± SDs. ns, no significant difference, **p < 0.01, ***p < 0.001

### High-dose loratadine promotes pyroptosis and interacts with apoptosis

Apoptotic signaling, which involves the activation of caspases, such as caspase-8 and caspase-3, can induce other types of cell death. Therefore, we administered a high dose of loratadine (twofold IC50, H-loratadine) to further investigate its effects. After 48 h of treatment, compared with the DMSO group, cells in the loratadine group displayed distinct membrane blebbing morphological changes when observed under a microscope (Fig. 3A). Colony formation with H-loratadine resulted in a variable effect, with over a 90% reduction in colony formation compared to the DMSO group (Fig. 3B). Flow cytometry analysis revealed that high doses of loratadine can induce pyroptosis, with a significantly increased proportion of Annexin V and PI double-positive staining in the high-dose loratadine group (70.667 ± 2.558%) compared to the control group (9.773 ± 1.315%), and the difference was statistically significant (*P* < 0.05) (Fig. 3C-F). The cleavage and activation of GSDMD or GSDME by inflammatory caspases, such as caspase-1, caspase-3, caspase-4 and caspase-8, can be assessed in the H-loratadine group by Western blot analysis. (Figs. 3J, I and 4E). During our investigation, we made a novel discovery that H-loratadine may stimulate the upregulation of PPARγ expression (Fig. 4F), which in turn acts as a promoter of GSDMD, as verified through a luciferase reporter assay (Fig. 4B**)**. Using ELISA to detect the concentration of IL-18 in the cell supernatant, it was found that the high loratadine group was linked to an elevated release of proinflammatory cytokines, and the difference was statistically significant (*P* < 0.001) (Fig. 3L).

To further investigate the crosstalk between apoptosis and pyroptosis, a range of pharmacological agents, including necrostatin-1, spautin-1, deferoxamine mesylate and disulfiram, were employed to investigate the potential occurrence of pyroptosis in H-loratadine-treated lung cancer cells. These compounds were utilized to discern whether cells undergo pyroptotic cell death. Additionally, a panel of caspase inhibitors, such as z-VAD-fmk, z-DEVD-fmk, z-IETD-fmk, ac-FLTD-cmk, and ac-YVAD-cmk, were used to assess their capacity to inhibit cell apoptosis or pyroptosis. In Fig. 4A, the ballooning-like cells were partially rescued by disulfiram but not by inhibitors of apoptosis (z-VAD-fmk), ferroptosis (deferoxamine mesylate), necroptosis (necrostatin-1) or autophagy (spautin-1). Western blot analysis presented in Fig. 4C-E demonstrated that caspase 3 inhibitor (z-DEVD-fmk), caspase 8 inhibitor (z-IETD-fmk), caspase 1/4/5 inhibitor (ac-FLTD-cmk, also known as GSDMD inhibitor) and caspase 1 inhibitor (ac-YVAD-cmk) can partially inhibit M-loratadine-induced apoptosis as well as H-loratadine-induced pyroptosis in A549 cells or H1299 cells. Our results contribute to a more profound exploration of the cell death mechanisms in loratadine-induced cell death.

### Loratadine treatment suppresses tumorigenesis and activates p53 and GSDMD in C57BL/6 mice

Piqued by the clinical and ex vivo findings, we sought to determine whether loratadine exhibits tumor-suppressive properties. Lewis cells (LLCs) were implanted in the right flank region of C57BL/6 mice, and metastatic colonization capacity was evaluated by assessing tumor nodule formation in the lungs after LLC tail vein injection. All models were administered loratadine or vehicle via intragastric administration. Tumor size measurements demonstrated that loratadine diminished tumor cell growth and metastasis in C57BL/6 mice in a dose-dependent manner (Fig. 5A-D). Moreover, the vascular morphology of mouse tumor sections was stained with DAPI and CD31 to evaluate the antivascularization ability of loratadine. Although the loratadine-treated group exhibited reduced microvessel density compared to vehicle-treated mice, this difference was not statistically significant in C57BL/6 mice (Fig. 5E-G). IHC staining verified a minor decrease in the levels of Ki67 with a significant increase in p53 and GSDMD staining in loratadine-treated LLC tumors (Fig. 5F and H-J). In conclusion, these studies indicate that loratadine may prevent tumors from growing, presumably through the regulation of proliferation via apoptotic and pyroptotic pathways as well as the regulation of the immune system.

## Discussion

A key finding of our study is that lung cancer patients receiving loratadine exhibit improved survival outcomes. Significant differences were observed in OS curves and PFS curves between patients with and without loratadine use. As the loratadine dose increased, patients experienced a significant improvement in outcomes. The results from animal experiments also conformed to the clinical outcomes, suggesting dose-dependent improvement. Our findings not only align with but also expand upon previous research. A study examining the impact of antihistamine use among Danish patients found that loratadine use was significantly linked to reduced all-cause mortality in patients with NSCLC or any cancer when compared to the usage of antihistamines that are not CAD [15]. Another nationwide Danish cohort also demonstrated that the use of antihistamines was related to a prognostic benefit in patients with ovarian cancer [17]. Furthermore, a recent study reported an association between improved survival in melanoma and breast cancer patients and the use of H1-antihistamines desloratadine and loratadine [18]. Recent retrospective analyses have shown that cancer patients who received antihistamines during immunotherapy treatment demonstrated dramatically enhanced survival outcomes [19].

M-loratadine have been found to influence a range of cellular pathways, notably those pertaining to the the cell cycle, cell senescence, P53 signaling pathway and apoptosis. Empirical evidence from scratch wound assays, colony formation assays, and Western blot analyses confirms that M-loratadine can concurrently induce cell senescence and apoptosis in addition to inhibiting epithelial-mesenchymal transition (EMT). This observation is in alignment with previous findings that suggest cell senescence and apoptosis may be hindered by the EMT process—a phenomenon that could enhance cellular survival during EMT [16]. Recent literature further substantiates a complex interconnection between apoptosis and senescence, indicating that both processes may be regulated by shared mitochondrial-dependent pathways [20]. In addition, these findings align with previous studies suggesting loratadine's antitumor effects through inhibiting the cell cycle or inducing "silent" cell death, such as apoptosis. For instance, loratadine may result in G2/M phase cell cycle arrest and death in colon cancer cells (COLO 205) [21]. Consistently, loratadine reverted multidrug resistance in NSCLC, breast and prostate cancer cells and sensitizes NSCLC cells to chemotherapy by inducing apoptotic and lysosomal cell death [15]. Loratadine may also directly damage DNA and activate Chk1, promoting G2/M arrest and making cells more susceptible to radiation-induced DNA damage while downregulating total Chk1 and Cyclin B [22]. Besides, researches on loratadine combination therapy also proved effective in cancer treatment. Loratadine, in combination with thioridazine may inhibit the rapamycin signaling pathway via phosphoinositide 3-kinase/Akt/mammalian target in gastrointestinal tumor [23] and effectively overcome immune evasion by suppressing CRC growth in a mouse model [24]. Moreover, H1-antihistamine treatment can enhance immunotherapy response via activation of the macrophage histamine receptor H1 [19].

Our study has uncovered some intriguing and novel findings. High-dose loratadine induced pyroptosis, a new form of cell death, alongside traditional apoptosis. Detailed analysis of cell models treated with high doses of loratadine and various inhibitors suggests a significant role for caspases in mediating both apoptotic and pyroptotic pathways. A critical discovery was that high-dose loratadine augmented the PPARγ level, which subsequently spurred GSDMD transcription by our luciferase reporter assay. This finding is further substantiated by reports in the literature indicating that PPARγ is instrumental in the regulation of caspase-8 activation [25]. Tumor sections from animal models, stained for Ki67 and GSDMD, reinforce the hypothesis that loratadine hampers tumor proliferation by promoting pyroptosis. Collectively, these results highlight a novel mechanism by which loratadine may exert anti-tumoral effects through the induction of pyroptosis.

The interplay between pyroptosis and apoptosis is intricate. They are two distinct forms of programmed cell death that are crucial for maintaining cellular homeostasis and responding to varying loratadine dosages. Caspase-8, a critical molecule that interconnects apoptosis and pyroptosis, can promote apoptosis via the extrinsic pathway and regulate pyroptosis by interacting with inflammasomes and modulating inflammatory caspases and gasdermin D (GSDMD) or gasdermin E (GSDME) activity by activating other caspases, such as caspase-1, caspase-4/5, and caspase-3 [26–29]. Various studies have emphasized the crosstalk between apoptosis and pyroptosis in different contexts. Orning et al. demonstrated that caspase-8 acts as a regulator of GSDMD-driven cell death and can cleave GSDMD during bacterial infection by Yersinia, leading to pyroptosis and the release of proinflammatory cytokines [26]. Sarhan et al. demonstrated that activating caspase-8 leads to the cleavage of both gasdermin D (GSDMD) and gasdermin E (GSDME) in macrophages, which results in pyroptosis. The absence of GsdmD causes a delay in membrane rupture, which in turn prevents the morphological changes associated with cell death from transitioning to apoptosis. This finding highlights caspase-8's dual role in modulating both apoptosis and pyroptosis, depending on the cellular context [27]. These studies underscore the importance of understanding the interplay between apoptosis and pyroptosis for cancer treatment, as targeting this crosstalk may provide new therapeutic opportunities for treating various cancers.

However, our investigation had certain limitations. First, the retrospective and single-center nature of the study might introduce unidentified biases that could affect our analysis. Additionally, our study did not sufficiently investigate the greater detail in crosstalk between apoptosis and pyroptosis.

**Fig. 6:**
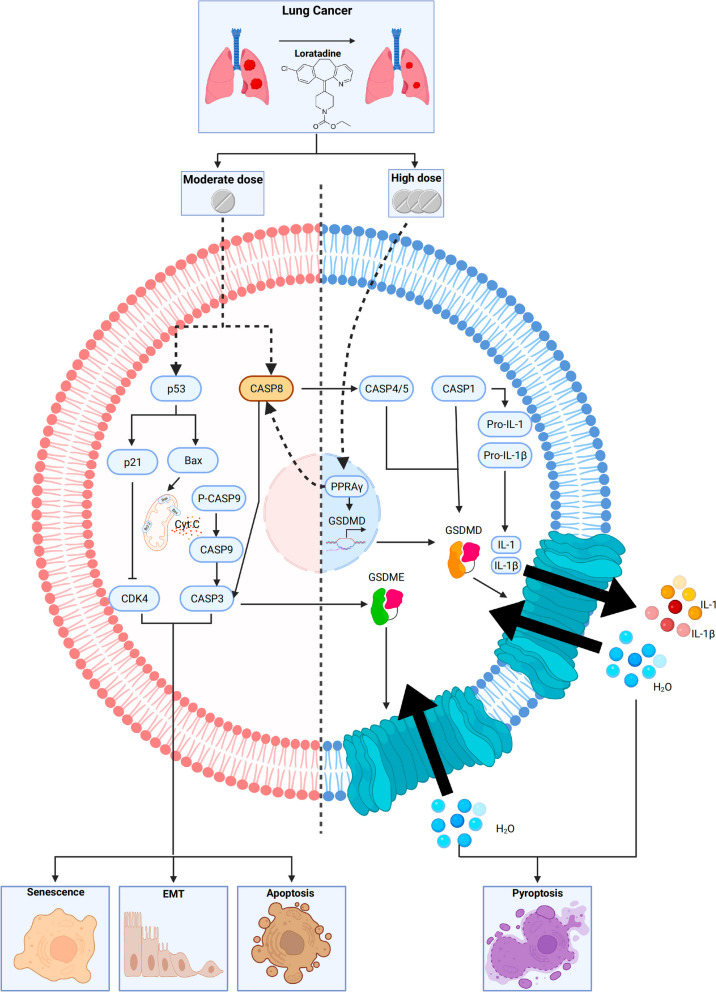
The impact of loratadine on lung cancer survival outcomes and underlying mechanisms. The administration of loratadine demonstrates a positive correlation with enhanced survival rates in individuals diagnosed with lung cancer. Loratadine could potentially foster complex crosstalk between apoptosis and pyroptosis, with caspase-8 playing a key role. This is achieved through the regulation of PPARγ levels, promoting gsdmd transcription and caspase-8 activation

In summary, we utilized a large case‒control population, virtually complete cancer ascertainment and histological verification, and detailed prescription data. The continuous updating of prescription data, along with accurate information on drug type and quantity, allowed for an extensive evaluation of exposure patterns and eliminated recall bias. Mechanistically, loratadine has the potential to incite a complex interplay between apoptosis and pyroptosis mediated by the crucial role of caspase-8. This is accomplished via the modulation of PPARγ levels that in turn stimulate gsdmd transcription and activate caspase-8 (summarized in Fig. 6). Further studies on any effects of other antihistamines or their immune response to tumors may also be merited.

## Data availability statement

The data that support the findings of this study are available from the corresponding author upon reasonable request.

### Supplementary Information

**Additional file 1.** 

**Additional file 2.** 

**Additional file 3.** 

**Additional file 4.** 

**Additional file 5.** 

**Additional file 6.** 

**Additional file 7. **Materials and Methods. 

**Additional file 8:** **Figure E1.** Hazard ratios of different combinations of loratadine and other drugs.

**Additional file 9:** **Figure E2.** IC50 values of lung cell lines.

**Additional file 10:** **Figure E3.** GSEA of DEGs. Several pathways and biological processes were differentially enriched, including negative regulation of the vascular endothelial cell proliferation signaling pathway. NES, normalized enrichment score; p.adj, adjusted P value; FDR, false discovery rate.

**Additional file 11:** **Figure E4.** KEGG pathway of cell senescence (hsa04218).

**Additional file 12:** **Figure E5.** Loratadine promotes senescence and apoptosis in vitro. (A) Loratadine-targeted gene expression. (B) GO enrichment categories of DEGs. (C) KEGG pathway (hsa04218). (D) KEGG pathway (hsa04110). (E) KEGG enrichment categories of DEGs. (F) KEGG pathway (hsa04115). (G) KEGG pathway (hsa04210).

## Abbreviations

LC: Lung Cancer
NSCLC: Non-small cell lung cancer
SCLC: Small cell lung cancer
OS: Overall survival
DFS: Disease-free survival
CTD: The Comparative Toxicogenomics Database
HR: Hazard ratio
GO: Gene Ontology enrichment
KEGG: Kyoto Encyclopedia of Genes and Genomes enrichment
GSEA: Gene set enrichment analysis
FDR: False discovery rate
NES: Normalized enrichment score
TSS: Transcription start sites
M-LOR: Moderate dose of loratadine
EMT: Epithelial-mesenchymal transition
H-LOR: High dose of loratadine
