# Supplementary material for: Loratidine is associated with improved prognosis and exerts antineoplastic effects via apoptotic and pyroptotic crosstalk in lung cancer
Source: J Exp Clin Cancer Res. 2024 Jan 2;43:5. doi: 10.1186/s13046-023-02914-8 (PMC10759632; doi:10.1186/s13046-023-02914-8)
Supplement: Supplementary file 7 — Additional file 7. Materials and Methods. [file 13046_2023_2914_MOESM7_ESM.docx]

**Materials and Methods**

**Clinical Cohorts**

This study investigated the medication profile and medical history of patients with lung cancer. We investigated the database of patients diagnosed with lung cancer at the First Affiliated Hospital of Guangzhou Medical University between 2006 and 2018. Outpatient and inpatient electronic medical records were accessed to analyze medication information (including the name, dosage and when it was prescribed), basic patient information (age, gender, marital status, smoking history), comorbidity (hypertension, pleural effusion, diabetes, liver disease, tuberculosis, chronic obstructive pulmonary disease, coronary artery disease, non-hemorrhagic gastrointestinal disease, renal disease, brain infarction, lung infection), and pathological information. (lung cancer type, stage, grade，etc.) The primary outcome was Overall survival (OS). The secondary outcome was Disease free Survival (DFS). Lung cancer mortality data were obtained from patients' in-hospital information records or follow-up by phone.

We compared different baseline characteristics of the patients according to mortality in our study. A Chi-square test performed to compare the categorical variables (age group, sex, marriage, comorbidities, pathology, and drug use). A t-test on continuous variables (age) was performed. We used a Cox proportional risk model and entered drug exposure as a time-dependent variable. First, we assessed the individual effects of different drugs on DFS and OS and adjusted for different covariates to identify independent associations between different drugs and the risk of lung cancer mortality: 1) model 1 was unadjusted, 2) model 2 was adjusted for sex, age, smoking, marital history, cancer type, degree of differentiation, and comorbidities, and 3) model 3 was based on model 2 and additionally adjusted for several other drug use. For the use of loratadine, further analyses were performed on its combination with nine other drugs, respectively. And the correlation of different cumulative doses of Loratadine (0 (no use), 60 mg, 60-120 mg, and >120 mg) and outcomes was analyzed. In sensitivity analyses, we stratified the analysis by administration of chemotherapy or not.

**Reagents**

Dimethyl sulfoxide (DMSO) was from Sigma-Aldrich. Necrostatin-1, spautin-1, and disulfiram were from Selleckchem. Deferoxamine mesylate was from MedChemExpress. The caspase inhibitors z-VAD-fmk, z-DEVD-fmk, z-IETD-fmk, ac-FLTD-cmk, and ac-YVAD-cmk were from Merck Millipore and MedChemExpress.

**Comparative Toxicogenomics Database**

The Comparative Toxicogenomics Database (http://ctdbase.org/,CTD)[1], which can effectively predict the correlation between diseases, drugs, and genes, was used to search for loratadine-related genes.

**Cell culture**

Human cell lines were purchased from Shanghai Institutes for Biological Sciences (Shanghai, China) as well as Procell (Wuhan, China). Cells were grown in DMEM (Life Technologies) or RPMI 1640 medium (Gibco) containing 10% FBS (ExCell Bio) and supplemented with 1% P/S (Gibico).

**Cytotoxicity assays**

For CCK8 (Cell Counting Kit-8) cytotoxicity assays, cells were seeded onto 96-well plates. After attaching to the plates’ surface, cells were treated with dimethyl sulfoxide (DMSO, MP Biomedicals) and a concentration gradient of loratadine: 0, 1.25, 2.5, 5, 10, 20, 40 and 80 µg/mL for 48 hours. Subsequently, cells were treated with CCK8 solution and incubated for an additional 2 hours according to the manufacturer's instructions (ApexBio). The optical density (OD) of each well at 450 nm was quantified on an BioTek Cytation 3 Imaging microplate Reader (BioTek Instruments).

**Wound-healing migration assay**

Cells were maintained in 24-well dishes till reaching 80% density, and scratched with 200 μl tips. After washing with PBS, cells were treated with indicated conditioned medium and photographed every 1 hour for 2 or 3days using Agilent BioTek Lionheart Automated Microscopes machine (https://www.biotek.com/).

Migrated distance was measures and analyzed by ImageJ software (NIH). Migration area = (pre migration area – migration area)/ pre migration area.

**Colony formation assay**

The 1000 cells were plated in a 6-well plate and allowed to attach at 37 °C with 5% CO2 for 12 h, then treated with loratadine. After 7 days of growth, colonies were fixed and stained with crystal violet, which was guided by clonogenic assay [2].

**Western blotting**

Concentration of protein lysates in cell extracts was determined with BCA assay (Beyotime Biotechnology), and 10 μg of cell extracts were loaded on 10% SDS-polyacrylamide gels and transferred to a 0.2-µm pore-size PVDF membrane. Membranes were then incubated with primary antibodies (The following antibodies were used: anti-GSDMD (Ab cam, ab210070,1:1000), anti-GSDME (Abcam, ab215191,1:1000), anti-β-actin (Beyotime Biotechnology, 1:3000), anti- pro Caspase-1 + p10 + p12 (Abcam, ab179515,1:1000), anti-Caspase-4 antibodies (Abcam,ab238124，1:1000), anti-Caspase-5 antibodies (Abcam, ab40887, 1:1000), anti-Caspase-3 antibodies (CELL SIGNALING TECHNOLOGY, #9662, 1:1000), anti-Caspase-8 antibodies (CELL SIGNALING TECHNOLOGY, #9496, 1:1000), anti-p21 antibodies (Abclonal, A1483, 1:1000), anti-p53 antibodies (Abclonal, A10610, 1:1000), anti-Bax antibodies (Abclonal, A19684，1:1000), anti-Bcl-2 antibodies (Sino biological, 101579-T32, 1:1000), anti-PARP antibodies (CELL SIGNALING TECHNOLOGY, #9541, 1:1000), anti-ASC antibodies (Abclonal, A1170, 1:1000), and anti-PPARγ antibodies (Abclonal, A11183, 1:1000)overnight. After washing in TBS containing 0.05% (v/v) Tween 20 (TBST), membranes were incubated with secondary antibodies (Beyotime Biotechnology,1:3000) for 1 hour at 37 ˚C and washed again. Finally, after incubation with Pierce ECL Plus western blotting substrate, images were acquired using a BioRAD chemiDoc MP Imaging System (BioRAD).

**Enrichment analysis**

Enrichment analysis including GO Enrichment and KEGG Enrichment was performed using the OmicShare tools, an online platform for data analysis ([https://www.omicshare.com/tools). Terms](https://www.omicshare.com/tools).%20Terms) with |logFC| >1 and padj <0.05 were considered as significance.

**RNA sequencing and analysis**

RNA sequencing was performed on A549 cell lines treated with different doses of loratadine, including a control group and a moderate dose group with an IC50 value. Total RNA was extracted 48 hours after treatment with either DMSO or the drug. The expression abundance (TPM) value of each gene was estimated, and differential expression was evaluated using cuffdiff. Only genes with a |fold change|> 2 and an adjusted p-value < 0.05 were considered to be statistically significantly differentially expressed between two groups. The BH false discovery rate correction was applied to the original p-value to obtain the adjusted p-value. This approach enabled the identification of differentially expressed genes with a high level of confidence. All sequencing data are available through the NCBI Sequence Read Archive under the accession number PRJNA961065.

**Gene set enrichment analysis (GSEA)**

GSEA was carried out by adopting the R package clusterProfiler (3.14.3) [3]to elucidate the pathway differences between the high- and low- NR0B2 groups. P-adj < 0.05, False discovery rate (FDR) < 0.25, and normalized enrichment score (|NES|) > 1 were considered as significance[4].

**Flow Cytometry**

A549 cells were exposed to either a moderate dose of loratadine (IC50), a high dose of loratadine (2-fold IC50), or DMSO for a duration of 48 hours. Following treatment, cells were washed and trypsinized before being stained with Annexin and PI using the FITC Annexin V Detection Kit I from BD Biosciences. Apoptosis or pyroptosis assessment was performed by measuring the percentage of live (annexin V-, PI-), necrotic (annexin V-, PI+), apoptotic (annexin V+, PI-), and pyroptotic (annexin V+, PI+) populations. This measurement was done using a BD Verse cell analyzer and FlowJo software (version 10.4.2; BD Life Sciences).

**ELISA analysis**

The presence of IL-18 in cell culture media supernatants was determined using the Human IL-18 ELISA Kit (Multisciences). Photometric measurements were taken at a wavelength of 450 nm using a microplate reader, specifically the Epoch (BioTek Instruments).

**Luciferase reporter assay**

Luciferase reporter assay was performed according to the manufacture’s manual (Yeasen Biotech, Dual Luciferase Reporter Gene Assay Kit). 0.1 μg of the desired plasmid (pGL3-Basic, pGL3-Promoter, pGL3-pGSDMD, or vecPPARg) was co-transfected with the control vector pRL 0.015μg by Hieff TransTM（Yeasen Biotech） into HEK-293 cells in a well of a 96 well plate. After 48 hr incubation, luciferase activity was measured with the Dual- GloMax luciferase assay system (Promega).

**In vivo tumorigenicity assay**

Animal experimental procedures were approved by the Laboratory Animal Use Management Committee of the Experimental Animal Institute of Guangzhou Medical University (approval number: 2021‐042,2021-167, 2022-223).

After adaptive feeding, 0.1mL of PC9 cell or lewis cell suspensions at a concentration of 0.5-1×106 cells/mL were subcutaneously injected into the right back of each C57B/L6 mouse. Besides, C57B/L6 mice were injected intravenously with lewis cells (5×10^5^) in the tail vein. Then, the mice with a tumor volume of 100 mm^3^ (average tumor volume of 100 mm3) or with tiny lung nodules were randomly divided into 4 groups, with five mice in each group. 0.1 mL vehicle, 5 mg/kg/d resveratrol, 35 mg/kg/d resveratrol, (or 50 mg/kg/d resveratrol) and 175 mg/kg/d resveratrol were inoculated with a feeding needle intragastrically directly.

**Tumor growth**

Tumor growth was weekly monitored using a digital caliper and the tumor volume was calculated by the formula (width)^2^ × length/2. Mice were euthanized and transplant-derived tumors or lungs were harvested when tumor size reached ∼ 1500 mm^3^ or when 14 days endpoint reached. The fixed tumor tissues were embedded in paraffin, sectioned, and stained with HE.

**Immunohistochemistry and immunofluorescence**

Immunohistochemistry was carried out on animal FFPE specimens. Antigen retrieval was performed by microwaving in citrate buffer (Thermo Scientific). Slides were incubated with anti-p53 antibody (ALPHA APPLIED, A19341, 1:5000), anti-Ki67 antibody (CELL SIGNALING TECHNOLOGY, #9129, 1:400), anti-CDK4 antibody (Abcam, ab108357,1:100), anti-GSDMD antibody (proteintech, 2077-1-AP, 1:75) as indicated.

For vessel density studies, tumor and normal tissues were used for anti-CD31 (Servicebio, GB113151, 1:100) immunofluorescence labeling and were calculated for the number of CD31 positive particles/total tissue area.

Immunolabelled slides were scanned using an 3DHistech digital slide scanner (Pannoramic MIDI) as well as NanoZoomer S360 (Hamamastu). All IHC staining images were quantified using ImageJ software (version 1.53k, NIH, USA) and the IHC profiler plugin.

**Micro-CT imaging and processing**

In vivo micro-CT scans was performed on mouse lung using a Bruker Skyscan 1276 micro-CT.

**Statistics.**

The data were presented as mean ± SD. To determine statistically significant differences between treatment and control groups, either a parametric unpaired t-test or a one-way ANOVA were utilized, accompanied by relevant post hoc tests. In cases where the data did not meet the assumptions of parametric tests, such as normal distribution, non-parametric statistical tests such as the Mann-Whitney rank sum test or Kruskal-Wallis test on ranks were used, followed by Dunn's test. Statistical calculations for P values were performed using SAS (version 9.4), GraphPad Prism (version 8.1.1) or R (version 4.2.1), and a P value <0.05 was considered statistically significant.

**Supplementary Reference**

1. Davis, A.P., et al., *Comparative Toxicogenomics Database (CTD): update 2021.* Nucleic Acids Res, 2021. **49**(D1): p. D1138-D1143.

2. Franken, N.A., et al., *Clonogenic assay of cells in vitro.* Nat Protoc, 2006. **1**(5): p. 2315-9.

3. Yu, G., et al., *clusterProfiler: an R package for comparing biological themes among gene clusters.* OMICS, 2012. **16**(5): p. 284-7.

4. Subramanian, A., et al., *Gene set enrichment analysis: a knowledge-based approach for interpreting genome-wide expression profiles.* Proc Natl Acad Sci U S A, 2005. **102**(43): p. 15545-50.
