# Supplementary figures and images for: Loratidine is associated with improved prognosis and exerts antineoplastic effects via apoptotic and pyroptotic crosstalk in lung cancer
Source: J Exp Clin Cancer Res. 2024 Jan 2;43:5. doi: 10.1186/s13046-023-02914-8 (PMC10759632; doi:10.1186/s13046-023-02914-8)

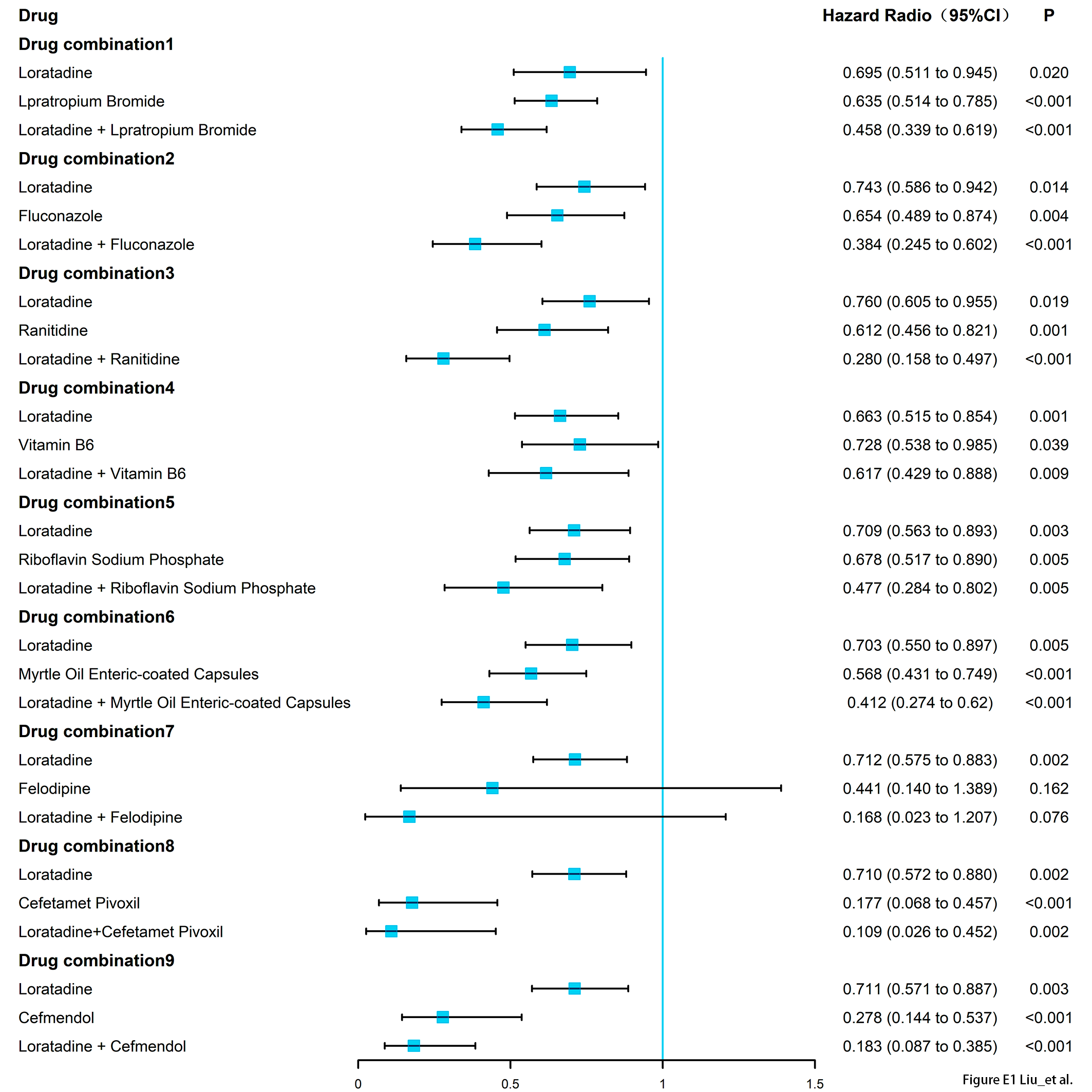

Supplement: Supplementary file 8 — Additional file 8: Figure E1. Hazard ratios of different combinations of loratadine and other drugs. [file 13046_2023_2914_MOESM8_ESM.tif]

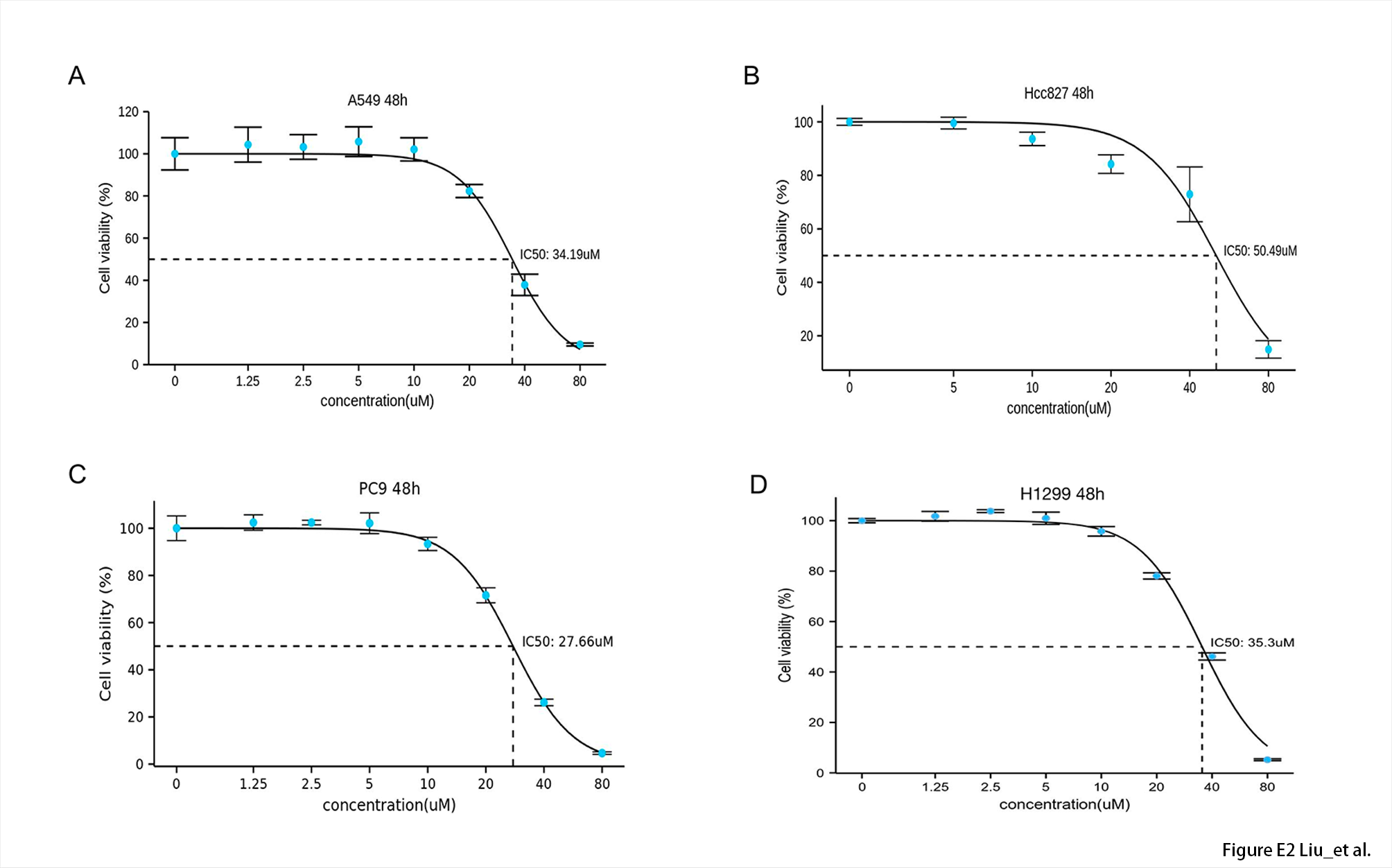

Supplement: Supplementary file 9 — Additional file 9: Figure E2. IC50 values of lung cell lines. [file 13046_2023_2914_MOESM9_ESM.tif]

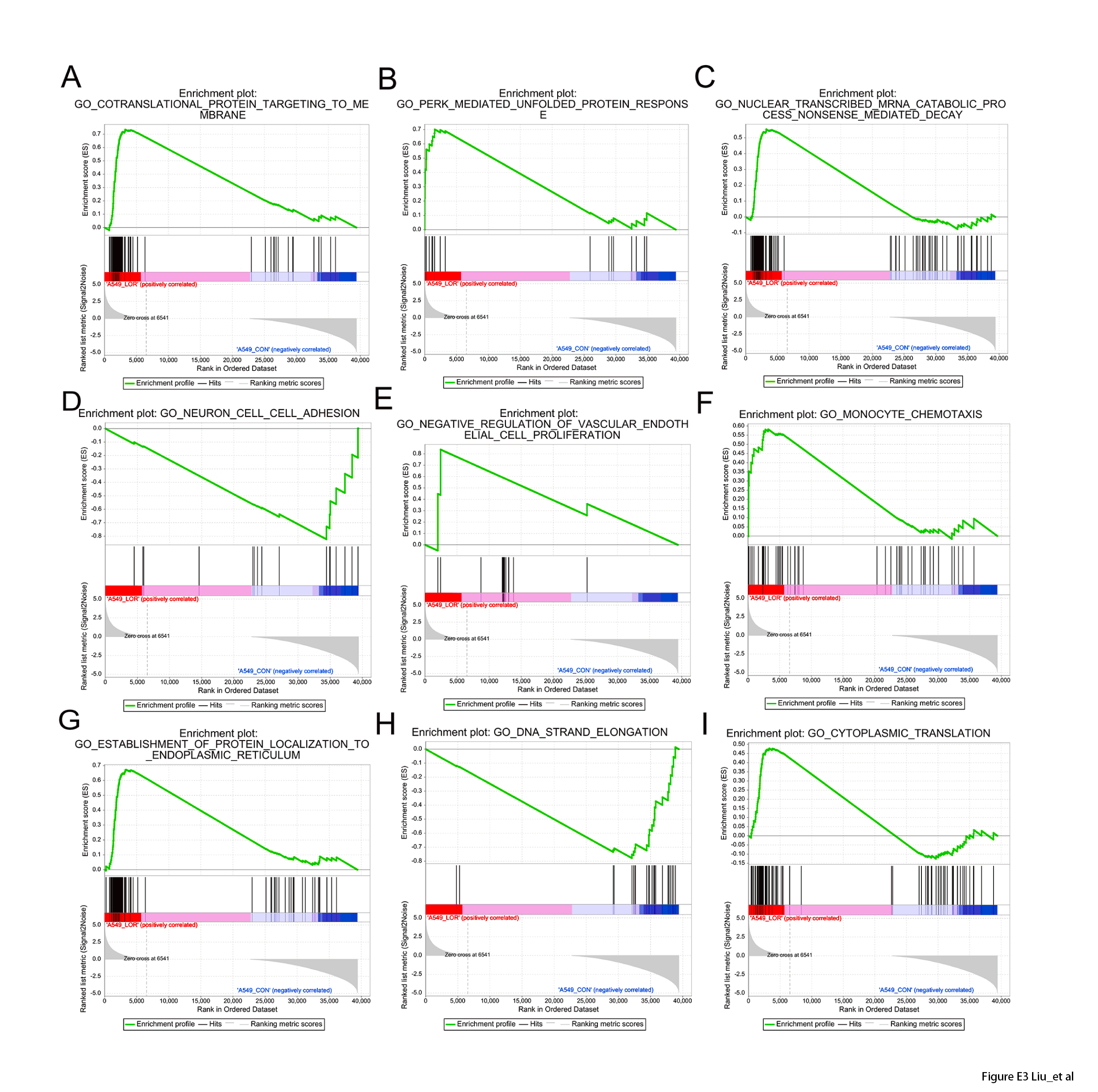

Supplement: Supplementary file 10 — Additional file 10: Figure E3. GSEA of DEGs. Several pathways and biological processes were differentially enriched, including negative regulation of the vascular endothelial cell proliferation signaling pathway. NES, normalized enrichment score; p.adj, adjusted P value; FDR, false discovery rate. [file 13046_2023_2914_MOESM10_ESM.tiff]

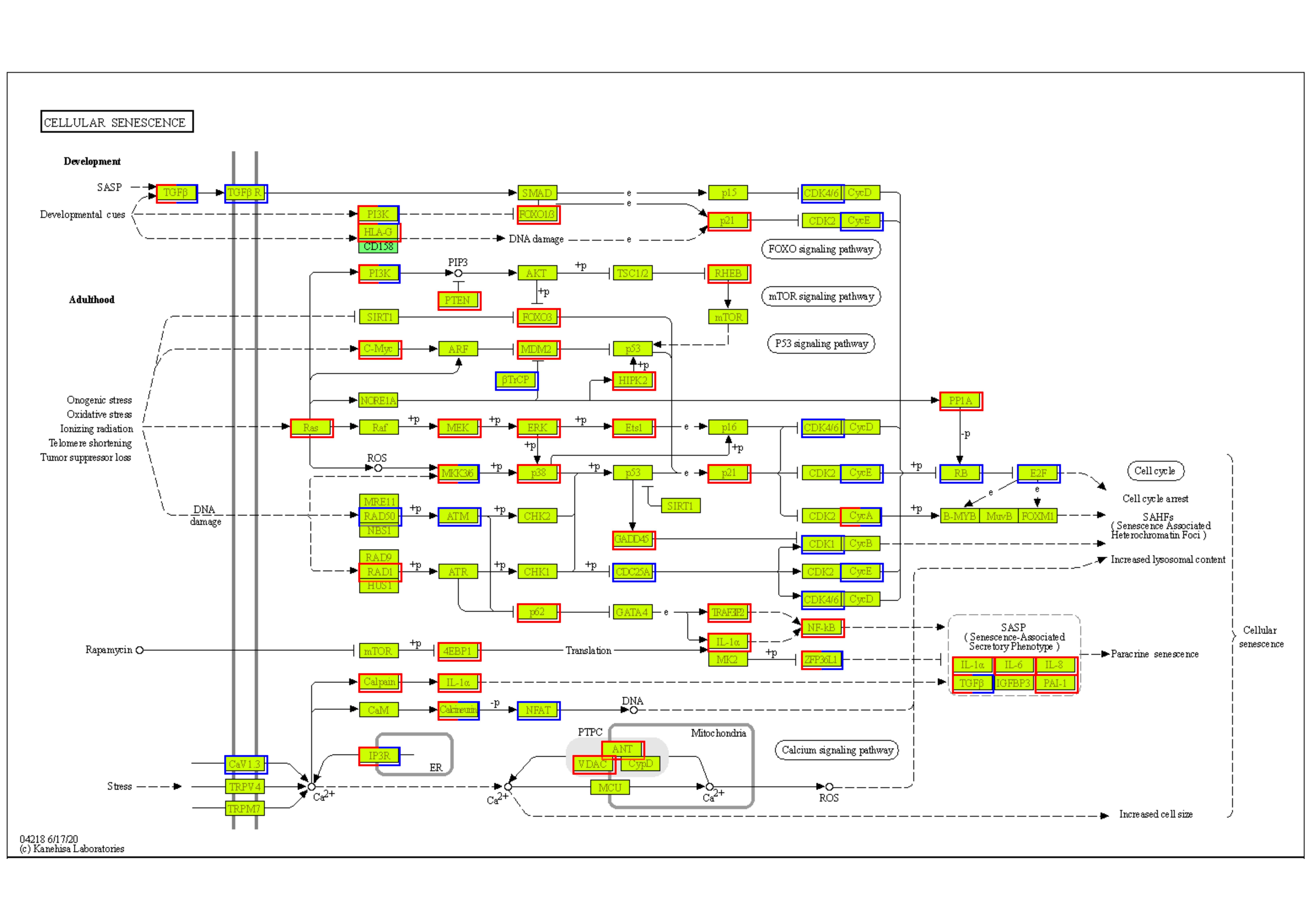

Supplement: Supplementary file 11 — Additional file 11: Figure E4. KEGG pathway of cell senescence (hsa04218). [file 13046_2023_2914_MOESM11_ESM.tiff]

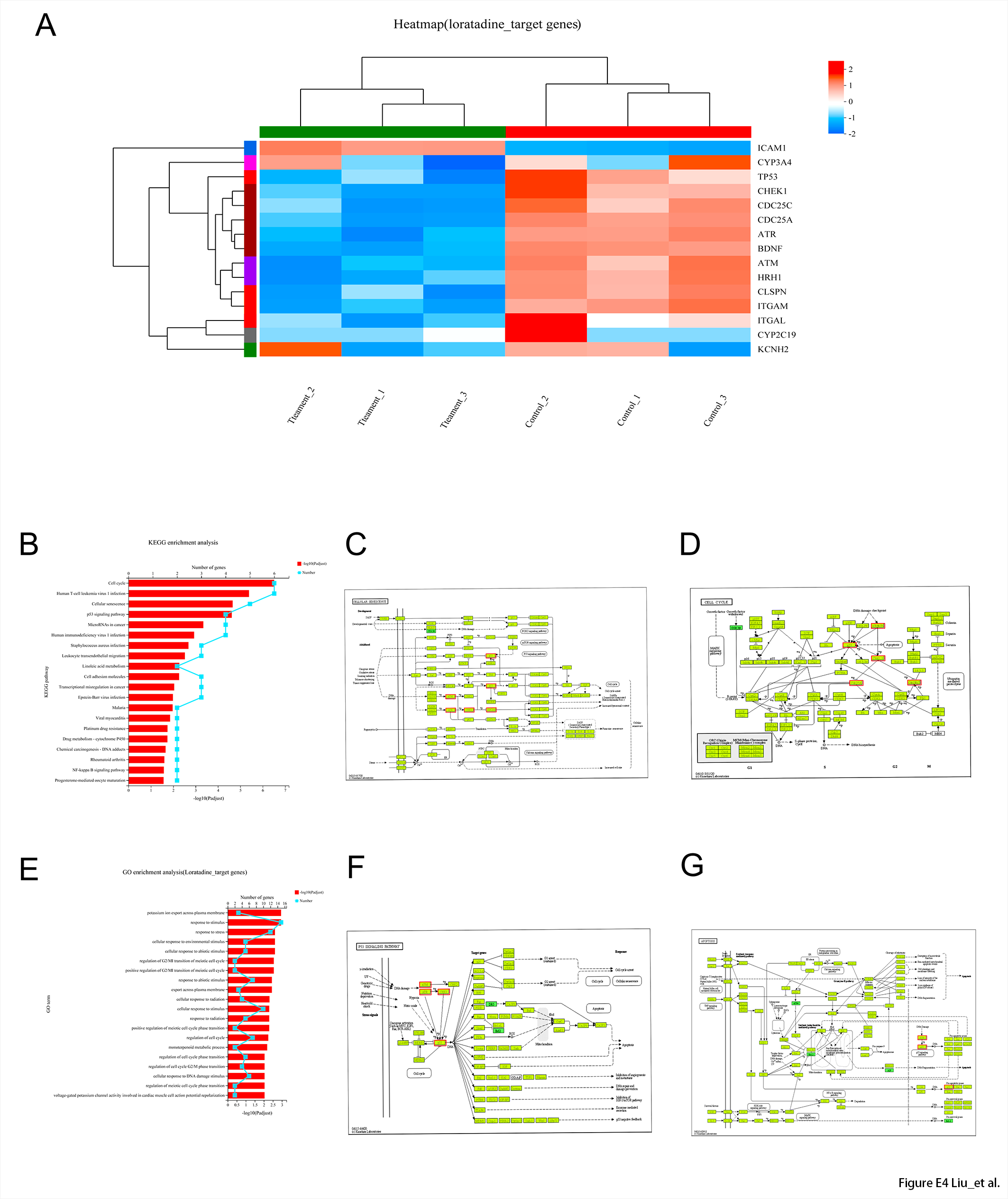

Supplement: Supplementary file 12 — Additional file 12: Figure E5. Loratadine promotes senescence and apoptosis in vitro. (A) Loratadine-targeted gene expression. (B) GO enrichment categories of DEGs. (C) KEGG pathway (hsa04218). (D) KEGG pathway (hsa04110). (E) KEGG enrichment categories of DEGs. (F) KEGG pathway (hsa04115). (G) KEGG pathway (hsa04210). [file 13046_2023_2914_MOESM12_ESM.tif]
